# Australian, Irish and Swedish women’s perceptions of what assisted them to breastfeed for six months: exploratory design using critical incident technique

**DOI:** 10.1186/s12889-016-3740-3

**Published:** 2016-10-10

**Authors:** Yvonne L. Hauck, Ingrid Blixt, Ingegerd Hildingsson, Louise Gallagher, Christine Rubertsson, Brooke Thomson, Lucy Lewis

**Affiliations:** 1 School of Nursing, Midwifery and Paramedicine, Curtin University, GPO Box U1987, Perth, WA 6845 Australia; 2 Department of Nursing and Midwifery Education and Research, King Edward Memorial Hospital, Bagot Rd, Subiaco, WA 6008 Australia; 3 Department of Women’s and Children’s Health, Uppsala University, 751 85 Uppsala, Sweden; 4 Centre for Clinical Research Sörmland, Uppsala University, Kungsgatan 41, 631 88 Eskilstuna, Sweden; 5 Trinity College Dublin, School of Nursing and Midwifery, 24 D’Olier Street, Dublin, Ireland

**Keywords:** Breastfeeding, Prevalence, International, Social support, Professional support, Self-efficacy

## Abstract

**Background:**

Breastfeeding initiation rates in some developed countries are high (98 % in Sweden and 96 % in Australia) whereas in others, they are not as favourable (46 % to 55 % in Ireland). Although the World Health Organization recommends exclusively breastfeeding for six months, 15 % of Australian women, 11 % of Swedish women and less than 7 % of Irish women achieve this goal. Awareness of what women in different countries perceive as essential breastfeeding support is a gap in our knowledge.

**Methods:**

Our aim was to explore Australian, Irish and Swedish women’s perceptions of what assisted them to continue breastfeeding for six months. An exploratory design using critical incident techniques was used. Recruitment occurred through advertisements in local newspapers and on social networking platforms. Initial sampling was purposive, followed by snowball sampling. Telephone interviews were conducted with 64 Irish, 139 Swedish and 153 Australian women who responded to one question “what has assisted you to continue breastfeeding for at least six months?” Content analysis was conducted and common categories determined to allow comparison of frequencies and priority ranking.

**Results:**

Categories reflected the individual mother, her inner social network, her outer social network (informal support either face to face or online), and societal support (health professionals, work environment and breastfeeding being regarded as the cultural norm). Categories ranked in the top five across the three countries were ‘informal face to face support’ and ‘maternal determination’. Swedish and Australian women ranked “health professional support” higher (first and third respectively) than Irish women who ranked ‘informal online support’ as second compared to ninth and tenth for Swedish and Australian women.

**Conclusions:**

The support required to assist breastfeeding women is complex and multi-faceted. Although common international categories were revealed, the ranking of these supportive categories varied. We must recognize how the cultural context of breastfeeding support can vary for women in differing countries and acknowledge the resourcefulness of women who embrace innovations such as social media where face to face formal and informal support are not as accessible.

## Background

Evidence from a systematic review confirmed the health benefits of breast milk, further supporting optimal duration for exclusive breastfeeding continuing to six months [1]. However, 37 % of infants less than six months are being exclusively breastfed in low and middle-income countries with even shorter duration in high-income countries [2].

Comparisons of ‘ever breastfed rates’ are promising for selected developed countries such as Sweden (98 %) and Australia (96 %), although for others rates are not as favourable (Ireland 46 %) [3]. Prevalence of ‘any breastfeeding’ at six months reflects declines with 72 % in Sweden and 60 % in Australia breastfeeding [3] with limited Irish data suggesting rates from 26 to 29 % [4, 5]. Variation in prevalence may reveal dissimilarity in how breastfeeding women are supported. For example, a comparison of ‘any breastfeeding’ for women residing rurally that found more Swedish women (88.3 %) were offering ‘any breastfeeding’ at two months compared to Australian women (75.8 %) [6]. Australian data collected in 2010 confirmed that 48 % of infants were exclusively breastfed at two months, 39 % at four months and 15 % at six months [4]. Swedish initiation rates were highest (98 %) with exclusive rates of 67 % and 51 % at two and four months, yet by six months only 11 % of Swedish infants were exclusively breastfed [7] compared to 15 % for Australian infants [8]. Irish national data reported that on discharge from hospital 55 % of women were offering ‘any breastfeeding’ and that by six months post birth less than 7 % were exclusively breastfeeding [4].

Factors associated with successful breastfeeding are multifaceted and include the internal or personal attributes of the woman and the formal support provided by health professionals and informal support from lay or peer groups [9]. Demographic characteristics such as maternal age, educational and income levels, and ethnicity have been found to influence breastfeeding practices. However, not all factors associated with initiation such as maternal age are associated with long term infant practices such as exclusive breastfeeding to six months [10]. Personality factors such as dispositional optimism, breastfeeding self-efficacy, faith in breast milk, breastfeeding expectations, intention and anxiety were associated with breastfeeding success [11, 12]. Informal support from a partner, mother and friends are also recognized as essential [11].

Research into interventions to support breastfeeding mothers has been extensive. Evidence reported in a systematic review has confirmed that peer counselling, lactation consultation and formal breastfeeding education during pregnancy have been found to increase duration [13]. In fact, attention to potential modifiable factors such as influencing breastfeeding intention, increasing self-efficacy and the provision of effective interventions around social support has been recommended for interventional studies [14]. Nonetheless, to be effective, support should ideally be tailored to the needs of the setting and population [9]. Strategies to support breastfeeding effective for one population in one setting may not be relevant or useful in other contexts. Although, countries may demonstrate promising initiation rates explanations for differing prevalence trends are not always apparent.

Achieving success with breastfeeding is complex. Although multiple layers of support may be available; we have limited knowledge as to what is most useful to women in differing contexts. Data from women’s perspectives as to what was helpful in their breastfeeding journey to six months in countries with high and lower prevalence rates can provide valuable information and also highlight factors which are potentially modifiable. This study addressed this gap in knowledge by exploring Australian, Irish and Swedish women’s perceptions and highlight how the context of support can differ between countries.

## Method

Our aim was to explore Australian, Irish and Swedish women’s perceptions of what assisted them to continue breastfeeding for at least six months. An exploratory design using critical incident techniques was used which allows for exploration of experiences and has been used to evaluate consumer expectations and perceptions in health care [15]. Use of this technique assumes that an incident can be clearly established, a detailed account is accessible and the incident is the basic unit of analysis [16]. For this study, the unit was the continuation of breastfeeding to six months. Critical incident techniques have been useful in the exploration of breastfeeding women’s perceptions of conflicting advice [17]. Rich data is collected from the participant’s perspective and in their own words. This method does not limit participants to a framework or forced choice responses as data is collected from interviews. Generally, up to 100 critical incidents are recommended [18]; however, final sample size is determined by data saturation. Human Research Ethics Committee approvals were obtained from Curtin University Human Research Ethics committee (No. SONM39-2014), School of Nursing and Midwifery Trinity College, Dublin and Regional Research and Ethics Committee of Uppsala University (No. 2015/285).

### Participants and data collection

Women, who breastfed a recent child for a minimum of six months, were invited to participate. Women could still be breastfeeding or have ceased within the past 12 months. To avoid recall bias, women currently breastfeeding or those who breastfed within the past 12 months were included as a period of three years or less has been recommended when focusing upon recall of infant feeding practices [19].

Australian recruitment occurred through advertisements in Community Newspapers or a Parents Paper, both freely available to consumers. The advertisements ran in March and April 2014. Participants confirmed interest through email or telephone. Swedish recruitment occurred through social media, social network Facebook and the three largest internet forums for parents in Sweden. Data were collected between October 2015 and January 2016. Irish recruitment was also through social media Facebook pages of breastfeeding groups and a popular parenting forum. Snowball sampling was them employed in all countries as mothers were encouraged to share study details with other women they knew who had recently breastfed for at least six months. Responding to advertisements and contacting the research team was considered ‘implied consent’. The researcher team made three attempts to contact women, whereby the study purpose was explained and the woman verbally confirmed her informed consent to participate in an audio-recorded telephone interview. Demographic data to describe participants from each country was collected prior to commencing the interview and included: maternal age, parity, maternal education, previous breastfeeding experience and feeding pattern at six months with their youngest child. Responses to one opened ended question “what has assisted you to continue breastfeeding for at least six months?” were recorded with the woman’s verbal permission. All participants answered the question and provided demographic data as no one withdrew from the study. During the interview a guide was used in order to facilitate data collection of demographic data, responses to the open ended question and priority ranking of responses shared by each woman. Prior to the interviews the guide was pilot tested for face validity with five Swedish, four Australian and four Irish women. Only minor wording formulations were made to capture the context in each country.

Using an open ended question rather than a tick box with predetermined factors is the novel aspect of this study. No assumptions were made as to the specific factors and the importance of these factors for these international women. After each interview member checking with each woman was used to ensure agreement of the summary of the interview as the interviewer provided a verbal summary of her responses which allowed the opportunity for reflection and to add anything she may have missed. Participants were then asked to rank the top three supportive features perceived as most important.

### Data analysis and storage

Content analysis was used to determine categories that women perceived were important for their continued breastfeeding. In summary, each interview was transcribed verbatim and read several times to get an impression of its content. Thereafter the text from the interviews were coded and sorted into categories which were discussed in the research groups across countries until a consensus agreement was reached.

During analysis, data was grouped into common categories that women from each country identified as being instrumental in assisting them to breastfeed for at least six months [20]. Researchers from each country shared their preliminary categories based upon analysis of transcripts and negotiation then occurred to determine final categories that reflected responses from Australian, Irish and Swedish women. Once agreement occurred with final categories, they were used to determine the citation frequency from women’s ranking of their importance. The responses Australian, Irish and Swedish women provided in their interviews were entered into separate SPSS databases and recoded to match the final categories agreed upon by the international research teams. Descriptive statistics were then calculated according to those ranked as first, second or third in assisting the woman to breastfeed for at least six months. De-identified transcript documents and demographic data will be securely stored on university password protected computers for seven to ten years depending upon country requirements.

## Results

Our participant profile included 153 Australian women, 64 Irish women and 139 Swedish women. Demographics summarized according to country are presented in Table 1. In general, women were: between 33 and 35 years of age; had an undergraduate or postgraduate degree; had one to two children (with their youngest child between 16 and 21 months); and were still offering breast milk (alone or with solid food) at the time of interview. Ten final categories were confirmed as representing the experiences of Australian, Irish and Swedish women. All categories are listed in Table 2. Each category will now be described with supportive quotes in italics acknowledged by a coding system (country and participant number) such as Aus43, Irish24 or Swed76.

**Table 1:** Demographic characteristics of Australian, Irish, and Swedish women

Variable	Australian women *N* = 153 n(%)	Irish women *N* = 64 n(%)	Swedish women *N* = 139 n(%)
^a^Maternal Age	33.5(4.92)[22–49]	34.9(4.01)[24–43]	33.5(5.61)[20–51]
^a^Number of children	1.7(0.83)[1–5]	1.6 (0.73)[1–4]	1.8(0.91)[1–6]
Number of children			
One child	75 (49.0)	31 (48.4)	62 (44.6)
Two children	55 (35.9)	25 (39.1)	51 (36.7)
Three children	18 (11.8)	7 (10.9)	21 (15.1)
Four or more children	5 (3.3)	1 (1.6)	5 (3.6)
Level of education			
High school not completed	2 (1.3)	-	5 (3.6)
High School completed	18 (11.8)	6 (9.4)	32 (23)
Undergraduate degree	88 (57.5)	28 (43.8)	62 (44.6)
Postgraduate degree	45 (29.4)	30 (46.9)	40 (28.8)
Previously breastfed a child	77 (50.3)	32 (50.0)	70 (50.4)
^a^Age of youngest child in months	16.7(9.75)[5–60)]	16.0(9.25)[6–42]	21.0(11.2)[6–56]
Youngest child feeding 6 months			
Breastmilk	21 (13.7)	17 (26.6)	30 (21.6)
Breastmilk and formula	1 (0.7)	1 (1.6)	4 (2.9)
Breastmilk and solid food	122 (79.7)	40 (62.5)	95 (68.3)
Breastmilk and formula and solid food	9 (5.9)	6 (9.4)	10 (7.2)

^a^Mean(Standard Deviation)[Range]

**Table 2:** International Categories: what assisted women to breastfed for six months

Maternal self-determination	
Maternal knowledge of health benefits	
Maternal awareness of psychological benefits	
Partner support	
Breastfeeding was going well	
Informal face to face support	
Informal online support	
Health professional support	
Work environment	
Cultural norm	

### Maternal self-determination

‘Maternal self-determination’, as a category, reflected comments shared by women around personal characteristics that contributed to persistence and determination. *You just have to be determined yourself, arm yourself with all the information you can and be super determined that you are going to succeed* (Irish58). First time mothers also expressed strong beliefs captured under this determination such as *breastfeeding comes with having a baby and formula feeding was not an option I considered* (Aus173) or *You have to know what you want and … be prepared to fight for it, it’s not as simple as just putting the baby to your breast* (Swed3). For some women, their resolve to breastfeed strengthened across the perinatal period: *If anyone had asked me before I would probably have said that I would breastfeed because it feels like a given for me, and then I got pregnant and then when I thought about it I was pretty sure I would breastfeed my baby* (Swe8). Some women suggested that their determination was influenced from a previous breastfeeding experience which could have been positive or negative. *I really regretted giving up at 4 months on my previous baby so I was absolutely determined to make it to at least 6mths with this baby, I said no matter what I’ll keep going and preserve this time around. I really believe in the benefit, I suppose that would be behind my determination* (Irish32).

### Maternal knowledge of health benefits

The second category ‘maternal knowledge of health benefits’ captured women’s statements around knowledge of the physiological benefits of breastfeeding including the provision of ideal nutrition and protection from antibodies for the infant: *For nutrition and the immune system…breastfeeding is good for the baby in general* (Swe27). Women acknowledged how they were well informed in their breastfeeding decision: *I knew it was beneficial to my baby’s growth and his immune system. He would be set up for life because I had no doubt that breast is best* (Aus34). A final quote supports how knowledgeable these women were: *I was really well read on the benefits and I totally believe that we should be telling everyone that breast milk and formula are just not the same. He has never been sick a day* (Irish24). This category also reflected women’s awareness of how breastfeeding could benefit the mother’s own health: *knowledge about breastfeeding health benefits for my baby and myself* (Aus182).

### Maternal awareness of psychological benefits

In addition to the physiological benefits women were also aware of how breastfeeding could facilitate bonding and feeling close to their infant. ‘Maternal awareness of psychological benefits’ is reflected in comments such as: *I absolutely loved every minute of it, the closeness with her [baby] was priceless, I felt super powered* (Irish33). The opportunity to facilitate closeness was expected: *I wanted to be able to bond with my baby and felt that breastfeeding would help with this* (Aus67). From women’s stories, it appears that many women did have this expectation met: *I think it has been really, really special with the closeness and I think we have developed a really good bond…it has been a really fantastic experience* (Swe22). Explaining the concept of closeness was challenging: *I find it so hard to explain the close bond you get from it…I’m not even sure that you can, I think it needs to be experienced……we just work as a wee unit me and him [baby]* (Irish20). Women with a history of bottle feeding were also able to differentiate how breastfeeding offered something unique: *It is really special to have a baby so close to you and the baby is in a way consuming your body, it makes it really special…the symbiosis that takes place…even though I also bottle-fed my second baby, I do think there is something special about breastfeeding* (Swe5).

### Partner support

The importance of ‘partner support’ was shared across all countries. The influence of the partner in a woman’s feeding decisions is obvious from comments such as: *If my partner had pushed me to use formula so that he could feed the baby as well and so on, if he had nagged me to do that it would of course have affected me* (Swe1). Examples of support included practical assistance such as: *He brought me drinks and food and made sure everything was in order so I could sit there with the baby in my arms and breastfeed and this made it possible for me to sit there and breastfeed peacefully* (Swe130). Partner support also acknowledged what breastfeeding meant to the woman: *My husband is just great, he totally gets how important it is to me and shares the workload 50/50. We had a rough start and he wasn’t keen on the co-sleeping at the start either but now he can see that he’ll get more sleep that way too (laughing)* (Irish54). Finally, acting as a champion when the woman was confronted by opposition was another role partners played: *My family were quite negative about it and asking ‘are you still at it’, only for him [partner] I’d have given up a lot sooner, his support for me was unwavering* (Irish28).

### Breastfeeding was going well

The fifth category, ‘breastfeeding was going well’ captures women’s comments around the ease and convenience of breastfeeding but also how the baby was thriving and enjoying breastfeeding, how their supply was ‘good’ and that they were able to express should they need to. *I know it’s not the same for everyone but I just love it and so does she [baby]… I just couldn’t imagine the hassle now of making up bottles. We went to Canada and back for a wedding and only had a few nappies and stuff to bring it was brilliant* (Irish27). The ease and convenience when breastfeeding was going well is captured in comments such as: *it’s* [breastfeeding] *just going so well for us both…I can’t think of a good reason to stop when it’s so easy and handy.* (Irish64) and *it was easy and convenient as I had a good supply* (Aus23). Women also considered that breastfeeding was going well based upon infant behavior: *baby settled well after feeding* (Aus25) and *She has chosen to breastfeed, it has calmed her if she has been unsettled so this has made me continue too. She seems to get full and she has done really well so far* (Swe25).

### Informal face to face support

‘Informal face to face support’ included support from peer counsellors, sisters, friends, cousins, grandmothers (maternal and paternal) and mothers’ group. Sometimes this support was readily available through organized mothers’ groups: *members of my mothers' group also breastfed* (Aus87) and *being in a community with like-minded mothers* (Aus151). However, other women had to actively seek this support: *There’s no group* [formal group run by a public health nurse] *in my area so I went out looking for help as soon as I got home and found La Leche League, thank God as I had really sore nipples and they truly saved the day…the leader* [Name] *rang me every day for a week to see how I was getting on* (Irish45). The support offered in these informal face to face connections could be practice advice or just the opportunity to talk to other women and not feel alone in their struggles. *Talking with other mothers and friends and sisters and just being able to talk about it because I think that I want to breastfeed, I’ve always wanted to breastfeed, but it still feels nice to be able to talk about how it’s actually quite difficult at times* (Swe24). It is apparent how this support can contribute to a mother’s determination and persistence to continue breastfeeding when challenges and difficulties arise: *I think primarily of my mother who has passed on her experiences and explained to me that it isn’t always that easy and she’s made me not give up when I’ve found the breastfeeding really hard* (Swe26). Informal social connections are not always supportive and women may selectively seek and embrace those whose support aligns with what the woman wants to assist with their breastfeeding efforts: *I had a few friends that had breastfed before me and had good and bad experiences so I would gravitate a lot towards those that aren’t negative about it. My own mother breastfed too so she was cool with it* (Irish59).

### Informal online support

The use of ‘informal online support’ was another category that reflected women’s use of social media [Facebook], and internet [chatrooms] to get the support they needed, particularly when informal face to face support was not readily available. Benefits of *being able to access an online breastfeeding group* (Aus131) and using *social media to see encouraging posts on breastfeeding* (Aus87) were acknowledged by two Australian mothers. Access to online support created a safe environment for women who could otherwise feel isolated: *I use the online a lot and I really value the other mums input on our Facebook page, we have our own little bubble and there’s no negativity or judgment* (Irish21). The value of this potential around the clock support is apparent from comments such as: *I’d never have continued without the online support, having people there at all hours of the night and day to answer all my questions is a huge support* (Irish8). Not only can this means of support offer knowledge and practical advice, *I read a lot and asked questions online to other parents before my second baby arrived – there were really, really knowledgeable people in that forum* (Swe6) but it contributes to women feeling emotionally acknowledged: *You help each other to motivate and support each other. Why it is good to continue – it is nice, it feels good to hear that what you are doing is right* (Swe66).

### Health professional support

The important support received from health professional along the breastfeeding journey was recognized. ‘Health professional support’ acknowledged midwives, child health or community nurses, lactation consultants, pediatric or neonatal nurses, nutritionists, and doctors. Although we focused upon breastfeeding for up to six months post birth, women did refer back to the impact of comments made during pregnancy: *She* [midwife] *talked a bit about the advantages of breastfeeding without putting pressure on me she said that she felt sorry for people who stopped breastfeeding too early and that it could be hard but it could be worth continuing* (Swe66). Others discussed the assistance received in hospital and how this got them off to a good start: *good support in hospital with lactation consultant* (Aus148). An Irish mother shared: *I had a real turning point at one week in the maternity hospital, I went in to see the lactation consultant and she talked … through everything. It was a total Eureka moment* (Irish35). The impact of one person on a woman’s breastfeeding experience cannot be underestimated as revealed by one Swedish mother:

*Then there was a nurse who said that we could give her some formula and this made me feel so useless because I didn’t want that and then we got to go home… then we came back and my milk production was fine and we met a really nice nurse who was an amazing support and she sent us home with the feeling that we would be able to do it without giving the baby formula, that we would be able to go home and just breastfeed her* (Swe138).

From women’s stories is appears that health professionals can have a profound influence on breastfeeding and as one Australian women noted how *getting the right advice* [from health professionals] *at right time especially from the 6 week to 3 month period* (Aus11) was essential to her breastfeeding success.

### Work environment

The ninth category ‘work environment’ refers to women’s stories of being able to stay at home, being financially supported with access to maternity leave, or having a supportive or flexible work environment were factors that contributed to their being able to breastfeed for six months. *The longer maternity leave makes a big difference you don’t have to be thinking about combining feeding and going back to work until they are well onto solids* (Irish49). One Australian mother shared how she has taken two years off work due to her ability to access maternity leave whereas another noted that her baby was located near her work environment and she *breastfed when I could in between work commitments* (Aus45). Obviously not all women have these options and acknowledge different work requirements: *I think I would have had problems with combining it with working – it’s maybe not everyone who can do both* (Swe29). In fact, many women commented on the preparation with work options they undertook to be able to breastfeed: *I went back to work earlier before and I really regret giving up so soon. This time I took the leave and the unpaid bit so it’s much easier to keep the feeding going* (Irish18).

### Cultural norm

The final category ‘cultural norm’ refers to how breastfeeding was regarded as the natural choice, that breastfeeding is what most people within this context would select given examples of a strong family history of breastfeeding and seeing this reinforced by important role models. *That my mother breastfed me… she breastfed my siblings too and I remember her sitting and breastfeeding them and this has probably influenced me a lot, what you get from home is what you think is the most natural* (Swe11). The women who participated in this study shared examples of what was regarded as ‘normal’: *was a normal thing to do in our family* (Aus70); *all of my family members breastfed* (Aus37); and *there was family expectation as everyone in my family has breastfed* (Aus2). Although Ireland’s breastfeeding prevalence rates are quite different to Sweden’s rates, comments from Irish women such as: *most of my friends breastfeed their babies and my family, in my immediate family circle it would be pretty normal and I think that gave me a positive outlook on breastfeeding even before I started* (Irish51) reflect a similar cultural norm. *Everyone has breastfed their children and fed them until they were maybe three… it has always been this way in our culture and it is very, very important to breastfeed so you get close to your child and for lots of other reasons* (Swe10).

### Ranking of categories by country

Although all ten categories were represented across stories from Australian, Irish and Swedish women, the ranking of their importance to mothers revealed similarities and differences between countries. The combined total categories ranked as first, second or third by women in each country are reported in Table 3. It must be remembered that all women interviewed had successfully breastfed a recent child for at least six months. Their ranking of these categories reflects their interpretation of how these categories contributed to their breastfeeding efforts within their social context. The only categories that were in the top five across countries were ‘informal face to face support’ and ‘maternal determination’. ‘Partner support’ was in the top three categories for Irish and Swedish women, whereas it was sixth for Australian women. ‘Informal online support’ ranked in the top two for Irish women but was ninth and tenth for Swedish and Australian women, respectively. ‘Health professional support’ was in the top three rankings for Swedish and Australian mothers, whereas Irish women ranked this as seventh. Categories ranked independently as first, second and third by women in Australia, Ireland and Sweden are presented in Appendices 4, 5 and 6.

**Table 3:** Total categories ranked first, second or third by Australian, Irish and Swedish women

Australian Category citations *N* = 449 n (%)		Irish Category citations *N* = 192 n (%)		Swedish Category citations *N* = 382 n (%)	
Breastfeeding was going well	100 (22.3)	Informal face to face support	41 (21.4)	Health professional support	55 (14.4)
Maternal knowledge of health benefits	89 (19.8)	Informal online support	30 (15.6)	Informal face to face support	47 (12.3)
Health professional support	66 (14.7)	Partner support	26 (13.5)	Partner support	47 (12.3)
Informal face to face support	49 (10.9)	Maternal self-determination	25 (13.0)	Maternal self-determination	44 (11.5)
Maternal self-determination	42 (9.3)	Breastfeeding was going well	21 (11.0)	Maternal knowledge of health benefits	41 (10.7)
Partner support	39 (8.7)	Maternal knowledge of health benefits	18 (9.4)	Breastfeeding was going well	41 (10.7)
Maternal awareness of psychological benefits	25 (5.6)	Health professional support	11 (5.7)	Maternal awareness of psychological benefits	40 (10.5)
Cultural norm	17 (3.8)	Cultural norm	9 (4.7)	Cultural norm	36 (9.4)
Work environment	14 (3.1)	Work environment	9 (4.7)	Informal online support	28 (7.3)
Informal online support	8 (1.8)	Maternal awareness of psychological benefits	2 (1.0)	Work environment	3 (0.8)
Total	449 (100)		192 (100)		382 (100)

## Discussion

The ten categories identified as assisting Australian, Irish and Swedish women to continue breastfeeding for six months, incorporate the individual (mother), inner social (partner and baby within immediate family), outer social (informal support either face to face or online), and societal support (health professionals, work environment and breastfeeding being regarded as the cultural norm). These categories fit within an ecological model highlighting factors that influence breastfeeding such as the mother/infant dyad, the family, the health care system, the community and societal/cultural factors [21]. Our findings align with the ecological model which highlights how multiple coordinated efforts through targeted interventions must incorporate all factors within an ecological model to effectively promote and support breastfeeding.

The support required to assist breastfeeding women is complex and multi-faceted. The promotion and support of breastfeeding is a collective societal responsibility as the world does not always provide a supportive and enabling environment for breastfeeding women [22]. Components of an enabling environment for breastfeeding includes ‘individual determinants’ including mother and infant attributes and mother-infant relationship; ‘setting determinants’ such as health systems and services, family and community and workplace and employment; and ‘structural determinants’ including social trends, advertising, and media. “Multifactorial determinants of breastfeeding need supportive measures at many levels, from legal and policy directives to social attitudes and values, women’s work and employment conditions, and health-care services to enable women to breastfeed” (p.491) [22].

All women in this international study cited how their knowledge of breastfeeding health benefits assisted in their efforts and reinforce the importance of knowledge dissemination through parent education and individual health professional consultations. Evidence must inform best practice in parent education, whether group or individual consultation [23]. Diligence must continue where knowledge can be shared with parents but also to address areas where knowledge may be lacking. For example, a Finish study found that prospective parents had deficiencies in knowledge around how to increase lactation, the sufficiency of breast milk in the first four months and within the context of hot summers, plus the management of alcohol consumption whilst breastfeeding [24].

Our category ‘breastfeeding was going well’ illustrates the importance of women receiving positive reassurance of their breastfeeding performance to build self-efficacy. Bandura who constructed the social cognitive theory claims that individuals need enough knowledge about behaviors affecting health but must also believe they have the capability for adoption of the behavior [25]. Individuals with self-efficacy believe they are capable of successfully performing certain behaviors [25]. As evidence suggests, self-efficacy can increase through mastery experience (past experience or successful initial attempts), verbal persuasion (trust and encouragement by a credible significant others), vicarious experience, or physiological and affective states [26–28]. Although our international participants acknowledge their own determination as important, constant struggles without some degree of success may be overwhelming. Women may experience difficulties during the early stages of breastfeeding and it is important that advice and support is timely. This support is particularly important in the early postpartum period as a longitudinal cohort study in Sweden found that 27 % of mothers had breastfeeding problems in the first month which was associated with early cessation [29]. In fact, an American study found that 60 % of women ceased breastfeeding earlier than desired and difficulties with lactation such as attachment issues; sore, cracked or bleeding nipples and pain [30].

Our international breastfeeding women acknowledged the importance of support from health professionals which is supported in the literature. An Australian mixed methods study reported that women relied on health professionals for advice and support revealing a theme of ‘not giving up despite difficulties’ [31]. Support from others who can encourage individuals to believe they have the ability to achieve what they seek (verbal persuasion) are especially important when struggling with difficulties [26, 27]. The extent and timeliness of services being able to offer early problem resolution when women are struggling in ‘those first few weeks’ is essential [31]. For example, effective breastfeeding technique is associated with increased breastfeeding self-efficacy [32] and demonstrates how Bandura’s mastery experience can increase breastfeeding self-efficacy [26, 27].

Irish women in our study did not rank health professional support as highly as Australian or Swedish women who ranked this support in their top three categories. Perhaps in an environment where this formal support is not readily accessible, these resourceful women used informal networks available either face to face through family, friends or their partner or through online media. Although Australian and Swedish women also cited partner and informal face to face contact within their top five rankings, use of informal online support were ninth and tenth, respectively. Evidence around the important of both informal and formal support has been widely known [33]. A recent UK study explored women’s experiences with Baby Café breastfeeding support groups and found that mothers valued a combination of professional and peer support provided by the Baby Care services: the social support from other mothers was regarded as central to being able to meet their breastfeeding goals [34].

The use of informal online support through social media such as Facebook was highly cited by Irish mothers. Bandura claimed that self-efficacy can increase through role models in social media and individuals can observe attitudes, styles of competencies and attainments of different people [26, 27] but limited evidence is available around the use of social media to support breastfeeding women. A Twitter based educational campaign on awareness, knowledge and breastfeeding practices in Saudi Arabia reported a slight increase in initiation with women confirming a willingness to continue exclusive breastfeeding (*n* = 484) [35]. A qualitative design explored the use of social media amongst 14 first time African American mothers and eight support persons [36]. Although acknowledged as an important vehicle to disseminate information, social media is not being used to its full potential and opportunities to create innovative, health interventions around infant feeding are not only recommended [36] but deemed essential for Generation Y who are online and connected [37].

Fathers and family members such as maternal grandmothers do influence a woman’s infant feeding decision [38, 39]. Although father presence is associated with greater initiation, a negative relationship between practical support from fathers and grandmothers and breastfeeding has been noted [40]. Fathers are acknowledged as providing essential support for their breastfeeding partners and initiatives to improve their knowledge around breastfeeding must continue to be a research priority as suggested from a pilot study with inner-city fathers in Ohio [41].

Awareness of the potential for clashes between idealism and the reality experienced within and between families and health professionals suggests that a family-centered narrative approach may be beneficial in acknowledging family goals particularly in the presence of breastfeeding problems [42]. Infant behaviors that can be misinterpreted as breastfeeding problems or perceptions of insufficient milk [43] and reflect misunderstanding of normal infant crying, an unsettled restless baby or frequent awakenings at night: it has been suggested that teaching child development to parents could be a useful strategy to extend breastfeeding duration [44].

The category of breastfeeding being noted as the cultural norm was similarly ranked eighth by all international women which is interesting given Australia and Sweden have high initiation rates and comparable prevalence up to 6 months. However, Irish women where initiation rates are lower also ranked cultural norm as eighth out of the ten categories. This highlights that acknowledging breastfeeding as a cultural norm was important for these international women who all breastfed to at least six months and whose personal and social environment reinforced the normality of breastfeeding. Individual’s everyday network may include important role models (vicarious experience) because they reinforce that individuals “just like you” can be successful and are essential for increased self-efficacy [27]. In fact, further research has been recommended across pre-conception and throughout the perinatal period to better support women choosing to breastfeed and cultivate breastfeeding as a cultural norm [45].

We found that mothers in this study cited felt that breastfeeding facilitated bonding and enabled them to feel closeness with their infant. Mothers enjoyed the closeness and therefore had a positive experience of breastfeeding. When individuals are happy, relaxed and calm (physiological and affective states) they may achieve higher self-efficacy [26–28]. It has been suggested that social and professional support should include help for mothers to relax and focus on their feelings of closeness with their infant [46].

Limitations must be considered when interpreting our findings as our Australian, Irish and Swedish women are not representative of all breastfeeding women in these countries. Women self-selected to participate in response to our recruitment strategies and represent a cohort of highly educated women who have continued to breastfed a recent child beyond six months. As social media platforms were used for recruitment in Sweden and Ireland, this may have contributed to the importance of online support in our findings.

## Conclusion

Our findings highlight Australian, Irish and Swedish women’s perceptions of what assisted them to breastfeed for six months suggesting that the support required to assist breastfeeding women is complex and multi-faceted. Although common categories were revealed, differences in the ranking of these supportive categories were noted between countries. A coordinated effort that recognizes all categories is recommended to support women’s breastfeeding efforts. Further intervention studies are warranted to explore appropriate support strategies within specific country contexts that can address women’s individual needs and intention to breastfeed thereby tailoring support for the needs of breastfeeding women in diverse countries.

## Appendix 4

**Table 4:** Categories ranked as first by Australian, Irish and Swedish women

Australian Categories *N* = 153 n (%)		Irish Categories *N* = 64 n (%)		Swedish Categories *N* = 139 n (%)	
Maternal knowledge of health benefits	54 (35.3)	Informal face to face support	13 (20.3)	Maternal self-determination	27 (19.4)
Health professional support	22 (14.4)	Partner support	11 (17.2)	Maternal knowledge of health benefits	21 (15.1)
Breastfeeding was going well	19 (12.4)	Informal online support	11 (17.2)	Maternal awareness of psychological benefits	21 (15.1)
Maternal self-determination	18 (11.8)	Maternal self-determination	8 (12.5)	Partner support	18 (12.9)
Partner support	17 (11.1)	Maternal knowledge of health benefits	7 (10.9)	Breastfeeding was going well	17 (12.2)
Informal face to face support	9 (5.9)	Breastfeeding was going well	5 (7.8)	Informal face to face support	11 (7.9)
Maternal awareness of psychological benefits	7 (4.6)	Cultural norm	4 (6.3)	Health professional support	10 (7.2)
Work environment	3 (2.0)	Work environment	4 (6.3)	Cultural norm	9 (6.5)
Cultural norm	3 (2.0)	Maternal awareness of psychological benefits	1 (1.6)	Informal online support	3 (2.2)
Informal online support	1 (0.7)	Health professional support	-	Work environment	2 (1.4)
Total	153 (100)		64 (100)		139 (100)

## Appendix 5

**Table 5:** Categories ranked as second by Australian, Irish and Swedish women

Australian Categories *N* = 153 n (%)		Irish Categories *N* = 64 n (%)		Swedish Categories *N* = 134 n (%)	
Breastfeeding was going well	41 (26.8)	Informal face to face support	13 (20.3)	Informal face to face support	22(16.4)
Maternal knowledge of health benefits	27 (17.6)	Partner support	10 (15.6)	Health professional support	20 (14.9)
Informal face to face support	21 (13.7)	Informal online support	9 (14.1)	Cultural norm	18 (13.4)
Health professional support	20 (13.1)	Breastfeeding was going well	8 (12.5)	Breastfeeding was going well	17 (12.7)
Maternal self-determination	12 (7.8)	Maternal knowledge of health benefits	7 (10.9)	Partner support	14 (10.4)
Partner support	11 (7.2)	Maternal self-determination	6 (9.4)	Informal online support	13 (9.7)
Cultural norm	8 (5.2)	Health professional support	6 (9.4)	Maternal awareness of psychological benefits	11 (8.2)
Maternal awareness of psychological benefits	6 (3.9)	Work environment	2 (3.1)	Maternal knowledge of health benefits	10 (7.5)
Work environment	4 (2.6)	Cultural norm	2 (3.1)	Maternal self-determination	9 (6.7)
Informal online support	3 (2.0)	Maternal awareness of psychological benefits	1 (1.6)	Work environment	-
Total	153 (100)		64 (100)		134 (100)

## Appendix 6

**Table 6:** Categories ranked as third by Australian, Irish and Swedish women

Australian Categories *N* = 143 n (%)		Irish Categories *N* = 64 n (%)		Swedish Categories *N* = 109 n (%)	
Breastfeeding was going well	40 (28.0)	Informal face to face support	15 (23.4)	Health professional support	25 (22.9)
Health professional support	24 (16.8)	Maternal self-determination	11 (17.2)	Partner support	15(13.8)
Informal face to face support	19 (13.3)	Informal online support	10 (15.6)	Informal face to face support	15 (13.8)
Maternal self-determination	12 (8.4)	Breastfeeding was going well	8 (12.5)	Informal online support	11 (10.1)
Maternal awareness of psychological benefits	12 (8.4)	Partner support	5 (7.8)	Maternal knowledge of health benefits	10 (9.2)
Partner support	11 (7.7)	Health professional support	5 (7.8)	Cultural norm	9 (8.3)
Maternal knowledge of health benefits	8 (5.6)	Maternal knowledge of health benefits	4 (6.3)	Maternal self-determination	8 (7.3)
Work environment	7 (4.9)	Work environment	3 (4.7)	Maternal awareness of psychological benefits	8 (7.3)
Cultural norm	6 (4.2)	Cultural norm	3 (4.7)	Breastfeeding was going well	7 (6.4)
Informal online support	4 (2.8)	Maternal awareness of psychological benefits	-	Work environment	1 (0.9)
Total	143 (100)		64 (100)		109 (100)
